# Comparison of Cell Arrays and Multi-Well Plates in Microscopy-Based Screening

**DOI:** 10.3390/ht7020013

**Published:** 2018-05-15

**Authors:** Ann-Kristin Becker, Holger Erfle, Manuel Gunkel, Nina Beil, Lars Kaderali, Vytaute Starkuviene

**Affiliations:** 1 Institute of Bioinformatics, University Medicine Greifswald, 17475 Greifswald, Germany; ann-kristin.becker@uni-greifswald.de; 2 BioQuant, Heidelberg University, 69120 Heidelberg, Germany; holger.erfle@bioquant.uni-heidelberg.de (H.E.); manuel.gunkel@bioquant.uni-heidelberg.de (M.G.); nina.beil@bioquant.uni-heidelberg.de (N.B.); 3 Institute of Biosciences, Vilnius University Life Sciences Center, LT-10257 Vilnius, Lithuania

**Keywords:** cell arrays, cell-based screening, RNA interference

## Abstract

Multi-well plates and cell arrays enable microscopy-based screening assays in which many samples can be analysed in parallel. Each of the formats possesses its own strengths and weaknesses, but reference comparisons between these platforms and their application rationale is lacking. We aim to fill this gap by comparing two RNA interference (RNAi)-mediated fluorescence microscopy-based assays, namely epidermal growth factor (EGF) internalization and cell cycle progression, on both platforms. Quantitative analysis revealed that both platforms enabled the generation of data with the appearance of the expected phenotypes significantly distinct from the negative controls. The measurements of cell cycle progression were less variable in multi-well plates. The result can largely be attributed to higher cell numbers resulting in less data variability when dealing with the assay generating phenotypic cell subpopulations. The EGF internalization assay with a uniform phenotype over nearly the whole cell population performed better on cell arrays than in multi-well plates. The result was achieved by scoring five times less cells on cell arrays than in multi-well plates, indicating the efficiency of the cell array format. Our data indicate that the choice of the screening platform primarily depends on the type of the cellular assay to achieve a maximum data quality and screen efficiency.

## 1. Introduction

Large-scale cell-based assays require platforms allowing many samples processed in parallel. The best-established formats are multi-well plates produced by numerous vendors (e.g., Greiner Bio One GmbH, Ibidi GmbH, Frisckenhausen, Germany) which are suitable for diverse applications in intact or lysed cells. Plates with 96- and 384-wells are used the most frequently, particularly for microscopy-based functional analysis screens, for example gene knock-down and, lately, knock-out experiments. Plates with 1536- and 3456-wells are also available on the market (e.g., Aurora Microplates Inc., Whitefish, MT, USA) with use being more common in chemical screens. 

Cell arrays, an alternative large-scale format which contains no physical sample separation was introduced by Ziauddin and Sabatini in 2001 [1]. In cell arrays, a transfection reagent and complementary DNA (cDNA), small interfering RNA (siRNA), microRNA (miRNA) or guide RNA (gRNA) are positioned in defined x- and y-coordinates by printing on a glass or plastic surface forming so called spots. The “ready-to-transfect” surface is then overlaid with cells which are settling on the surface and are transfected in the spot area. In this format, individual spots replace individual wells with each spot being a unique experiment. Lack of wells provides the possibility of scaling up and, indeed, up to several thousand spots can be analysed in parallel on a single cell array [2,3]. In contrast to multi-well plates, only few commercial products based on cell arrays are available. Both formats have their pros and cons which are summarized in the Table 1 and presented in more details below.

One of the major drawbacks of multi-well plates is that only middle scale of samples (24, 96 or 384 samples) can be analysed in one experiment. Plates with 1536- and 3456-wells are suitable to intact-cell-based screening only to a limited extend due to difficulties in cultivating cells and handling small amounts of the liquids in such formats. As a result, many plates need to be used when performing genome-scale screens. This increases time and costs of the experiment, and the variability of measurements due to between-plate-effects. Also, automated pipetting robots become indispensable. The multi-well plate format, in addition, suffers from the inefficient removal of reagents from the wells and difficulties with multiple washings. As a result, complex cellular assays which require multiple liquid exchange steps cannot be performed or need to be done in a simplified form only which might compromise the biological value of results. Finally, one of the key disadvantages of multi-well plates are edge-effects appearing due to evaporation of liquid from individual wells which is more prominent towards the outer edges. Many of these problems can be solved by using cell arrays which are compatible with complex cellular assays [4,5,6,7], and the problems related to liquid evaporation are nearly completely abolished.

On the other hand, cell arrays are limited in several applications, such as assays of cell migration and invasion or working with neuronal cells. Cell arrays are mainly compatible with reverse transfection—cells are added onto the pre-immobilized transfection complexes. A direct transfection on cell arrays—addition of transfection complexes on to the pre-seeded cells—could be possible by “sandwiching” procedures [8], but that requires additional robotics. Also, cell arrays bear a potential risk of sample cross-contamination. Several approaches can be used to prevent or minimize this risk: from fibronectin addition to the spotting solution which strengthens cell adhesion specifically to the spot area [1,9] to different surface coatings that prevent cell adhesion or liquid spreading between spots [3,10]. Currently, cell arrays are rarely used for chemical screenings due to difficulties in reliable and uniform immobilisation of chemical compounds in the spots; however, the applications in this area are emerging with some modifications in the cell array design [11]. In contrast, a clear strength of multi-well plates is that each well is physically isolated from the next. The possibility of reagent intermixing is limited by the well wall. Consequently, multi-well plates are well-established platform for chemical screening. Moreover, both, direct and reverse transfection can be performed on multi-well plates essentially with the same equipment.

Both platforms may be used for the cultivation of cells in 3D [10,12]. Also, both platforms can be used for genetics screens, be it clustered regularly interspaced short palindromic repeats (CRISPR)-Cas9 mediated gene knock-outs [13] or gene down-regulation via RNA interference(RNAi). Although the technology of RNAi has suffered from a number of critical issues, such as off-targets and poor reproducibility in the past [14], it is still a valid and widely used technology. For instance, RNAi screening-related data resulted in more than 50 PubMed cited publication in 2017. A number of comparative gene knock-out and knock-down screens were performed so far, revealing the complementary nature of the hits and deeper understanding of the underlying biology [15]. Here, we set out to compare data which are generated in both formats in two well-established assays, namely block of cell cycle progression and internalization of EGF (epidermal growth factor) using RNAi. We present and discuss the advantages and pitfalls of every system which potentially could be applied to the rapidly advancing large-scale gene editing-based studies.

## 2. Materials and Methods

### 2.1. Cell Culture and Materials

HeLa Kyoto cells have the accession number CVCL_1922 and were obtained from Dr Pepperkok, EMBL, Heidelberg, Germany. The cells were cultivated in Dulbecco’s modified Eagle’s medium (DMEM) (Gibco/Thermo Fischer Scientific, Waltham, MA, USA)supplemented with 5% (*v*/*v*) heat-inactivated foetal calf serum (FCS) (Biochrom-Merck, Berlin, Germany), 2 mM glutamine, 100 U/mL penicillin and 100 μg/mL streptomycin (Thermo Fischer Scientific, Waltham, MA, USA) in a humidified atmosphere with 5% CO_2_ at 37 °C. Silencer Select^®^ siRNAs targeting EGFR (s563, s564 s565), KIF11 (s7902), INCENP (s7423), PLK1 (s448), negative control No. 1 and Cy3 negative control No. 1 were purchased from Ambion (Thermo Fisher, Waltham, MA, USA).

### 2.2. Preparation of on Cell Arrays for Small Interfering RNA Transfection

OptiMEM (4.75 μL) (Thermo Fischer Scientific, Waltham, MA, USA) containing 0.4 M sucrose (USB, Cleveland, OH, USA), as well as 1.75 μL Lipofectamine 2000 (Thermo Fischer Scientific, Waltham, MA, USA) was transferred to each well of a 384-well low volume plate (BD Falcon, Widdington, UK) by hand. Afterwards, 5 μL of 3 μM siRNA solution were added and gently mixed. After incubation for 30 min at room temperature (RT), 7.25 μL of a 0.2% (*w*/*v*) gelatine solution containing 1% (*v*/*v*) fibronectin (both Sigma-Aldrich, St. Louis, MO, USA) were added and gently mixed. The transfection-mix was spotted by contact printing onto Thermo Scientific™ Nunc™ Lab-Tek™ chamber slides (Thermo Fischer Scientific, Waltham, MA, USA) using a BioRad Chipwriter Pro (BioRad, Hercules, CA, USA) equipped with 265 μm solid pins, resulting in a spot diameter of approximately 300 μm for all experiments. The spot volume was approximately 3 nL. 

### 2.3. Coating of Multi-Well Plates with Small Interfering RNAs

OptiMEM containing 0.4 M sucrose and a 0.2% solution of gelatine containing 1% fibronectin were prepared. Sucrose containing 4.75 μL of OptiMEM was mixed with 1.75 μL of Lipofectamine 2000 transfection reagent; 5 μL of 3 μM siRNA solution (unlabelled) and 0.5 μL of 3 μM siRNA-Cy3 solution were added and the mix incubated for 30 min at RT. Volume of 7.25 μL of 0.2% gelatine and fibronectin were added, diluted with milli-Q water (1:10), and distributed 5 μL to each well of the 384-well plate or 25 μL to each well of a 96-well plate (Ibidi GmbH, Planneg, Germany).

### 2.4. Cell Cycle Progression Assay

Cells (2000) in 80 μL growth medium were seeded on siRNA coated 384-well plates. 2 × 10^6^ cells in 20 mL growth medium were distributed with a pipette on cell arrays. In both formats cells were incubated for 24–30 h and then cells were fixed with 3% paraformaldehyde solution containing 0.25 ng/μL Hoechst-33342 (Thermo Fischer Scientific, Waltham, MA, USA) overnight at 4 °C.

### 2.5. Epidermal Growth Factor Internalization Assay

Cells (6000) in 150 μL growth medium were seeded in 96-well plates coated with the respective siRNAs. Cell seeding on cell arrays was done in the same way as for the assay of cell cycle progression. Cells were incubated for 36 h on both platforms and serum-starved for 12 h before the assay in DMEM with 0.01% (w/v) bovine serum albumin (BSA) (Carl Roth GmbH, Karlsruhe, Germany). Alexa Fluor 488 EGF complex (Thermo Fischer Scientific, Waltham, MA, USA) was added to DMEM-BSA medium to a final concentration of 100 ng/mL, cells were incubated at 37 °C for 20 min and fixed with 3% paraformaldehyde (PFA) for 20 min at RT. Hoechst-33342 was used to stain cell nuclei.

### 2.6. Microscopy

Multi-well plates were imaged using an Olympus IX81 ScanR system (Olympus Corporation, Tokyo, Japan) with a 20× magnifying objective. Cell arrays were imaged on an automated Olympus IX81 system (controlled via Micromanager/Python (https://micro-manager.org)) with a 20×magnifying objective using a Hamamatsu ORCA-FLASH 4.0LT camera (Hamamatsu Photonics Deutschland GmbH, Herrsching am Ammersee, Germany). Nuclei were recorded by using 350 nm peak excitation and 451 nm peak emission and internalized EGF-Alexa 488 was recorded using 488 nm peak excitation and 520 nm peak emission wavelengths. Alignment of the samples for the automated imaging was performed by referencing on the top left well/spot position and the known well-to-well or spot-to-spot distances. We imaged 25 subpositions for 96-well plates and 9 subpositions for 384-well plates in each well. On the cell arrays one image/spot was recorded. 

### 2.7. Image Analysis

**Figure 1:**
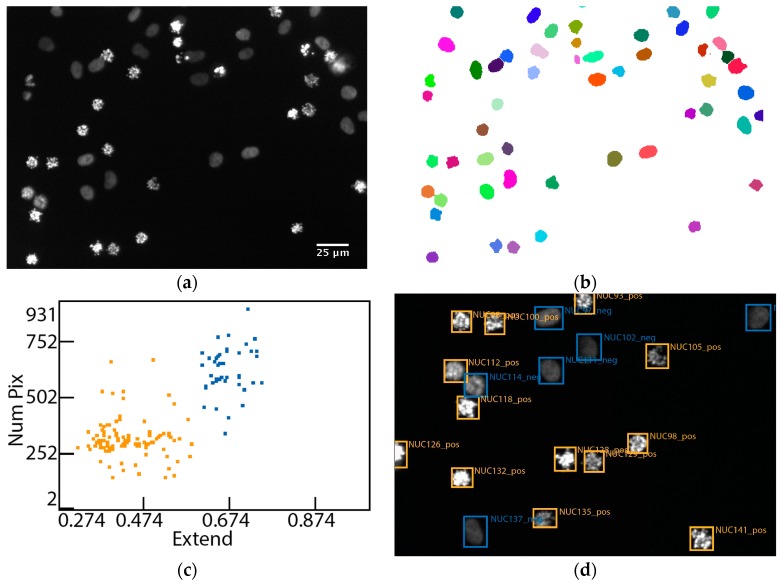
Classification scheme of RNA interference (RNAi)-induced nuclei changes. Within the microscope images (**a**), individual nuclei are segmented (**b**). Features of these nuclei are extracted upon which they can be classified, shown exemplary for the features “Num Pix” and “Extend” (**c**). An image excerpt with “spindle” phenotypes is shown (**d**) with the classification info shown as overlay in the image. Blue colour indicates normal interphase nuclei and yellow colour indicates nuclei with the spindle phenotype in (**c,d**).

Acquired images from the cell cycle assay were analysed for phenotypic classification with the Konstanz Information MinEr (www.knime.org, KNIME, Konstanz, Germany) and the KNIME Image Processing Plugin (KNIP) [16]. In order to identify the individual nuclei within the images, background was removed by applying a rolling ball background subtraction [17], followed by local and global thresholding steps in order to separate nuclei from the background. In our case, a rolling ball radius of 50 pixels was used, along with Otsu’s method for automatic global and the mean method for automatic local thresholding [18,19]. These steps are analogous to standard image processing steps as performed for instance by using Fiji or ImageJ [20]. Afterwards, morphological image operations are performed resulting in binary images, in which the nuclei are white and the background is black. Associated white regions in these binary images representing individual nuclei were identified by connected component analysis [21] (see also Figure 1) and the images were segmented based on this information. Morphological, textural and statistical features were extracted for each recognized nucleus. Based on these features the nuclei were classified as “normal nuclei”, “spindle phenotype” and “cytokinesis phenotype” based on the feature distribution of training data. The ratio of phenotypic objects in each subposition and within each well and on each spot was determined as readout. The KNIME workflow for this analysis is available online via github (https://github.com/manugunkel/KNIME-cellular-phenotyping). Epidermal growth factor internalization was analysed using MATLAB (https://es.mathworks.com/products/matlab.html, The MathWorks, Inc., Natick, MA, USA.). Nuclei were identified by thresholding and segmenting the images. Each segment was extended morphologically in order to obtain a surrounding area around the nuclei in which the mean EGF-Alexa 488 signal intensity was measured. 

### 2.8. Statistical Data Analysis

Statistical data analysis was done using R (http://www.r-project.org,) [22]. We used one-way analysis of variance (ANOVA) to test for statistical differences between means of different groups, taking unequal variances into account (oneway.test from R stats package). Post-hoc testing was done using the Games-Howell test, as this test is suitable for heteroscedastic data and provides robust results even for small sample sizes. Pearson correlation was computed to assess correlation between platforms. Lastly, we used Bartlett’s test of homogeneity of variances (bartlett.test) and the F-Test (var.test) to assess differences in variances, taking into account the possibly different sample sizes.

## 3. Results

### 3.1. Set-Up of the Assay to Quantify the Arrest of Cell Cycle Progression

Cell division in eukaryotes occurs during mitosis, which is a complex and highly regulated process. It consists of a series of events: prophase, metaphase, anaphase, telophase and cytokinesis, each of them having characteristic features and molecular regulators [23]. We explore this knowledge here to induce cell cycle arrest by RNAi-mediated down-regulation of three different regulators of mitosis: KIF11, INCENP and PLK1. KIF11 (Eg5) is a microtubule-based plus-end molecular motor which is required for establishing a bipolar mitotic spindle and separation of duplicated chromosomes [24]. Blocking of KIF11 function by siRNAs, antibodies or chemical inhibitors prevents centrosome migration and arrest cells in mitosis with a monopolar mitotic spindle [25,26]—called the “spindle” phenotype in this study (Figure 1). A similar phenotype is induced by RNAi knockdown of PLK1—serine/threonine polo-like kinase 1, which is implicated in the centrosomal localization of numerous mitotic regulators [27]. In particular, a proper localization and thereby function of three key mitotic kinases, namely, Aurora B, BUBR1 and Haspi is regulated via PLK1 [28]. The third control protein used in this study is INCENP—an inner centromere scaffold protein. It interacts to numerous other proteins required for orchestration of cell division events [29,30,31,32]. Depletion of INCENP induces defects in separation of the sister chromatids and cytokinesis with the appearance of multi- or binucleated cells [6], called the “cytokinesis” phenotype in this study. 

**Figure 2:**
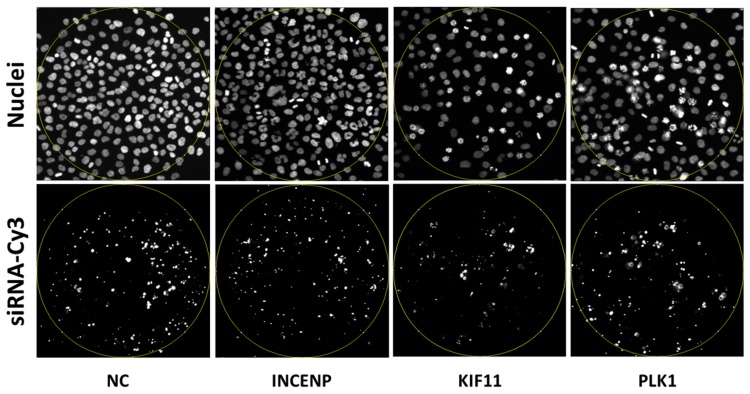
Spots on the cell arrays. In the upper panel, the nuclei phenotypes are shown following the co-transfection with the respective small interfering (siRNAs) and negative control (NC) siRNA labelled with Cy3 (lower panel). Yellow rings indicate the position of the spots.

RNA interference of KIF11, PLK1 and INCENP are widely used to assess the transfection efficiency in rapidly dividing cells, such as HeLa. Already 24–30 h following the transfection, a substantial sub-fraction of cells is arrested in their mitotic progression (Tables S1 and S2). In addition, the distorted mitosis can be unambiguously recognized by automated image processing and enables efficient quantification of the selected phenotypes. Previously tested and validated siRNAs were used to down-regulate each gene (see Section 2). Each siRNA was spotted nine times on the array with consequently nine images collected/per siRNA in one array. For validating the accuracy of the printing process and data acquisition, we have mixed fluorescently labelled siRNAs to the spotting solution (Figure 2). The position and size of the labelled spot within the image enables easy creation of the mask (region of interest) for the following data analysis. On average 156 HeLa cells/spot or over 1400 cells/siRNA/replica could be analysed. The final result of three independent replicas could be collected from as much as over 4000 cells for a single siRNA. Positioning of each spot/siRNA was identical for every siRNA used (Table S1). The data were collected using the arrays which were fabricated at the same time, but overlaid with cells at different days—three independent replicas were generated that way. Coating of 384-well plates was done in parallel to the spotting of the arrays to exclude possible variation among different batches of the reagents. Here, we also adhered to three independent replicas. We coated 6 wells with the same siRNA and 9 subpositions/well were imaged with 54 images/siRNA collected in one multi-well plate (Tables S3 and S4). Here, the numbers of cells were considerably higher: on average 1600 cells/well and 9600 cells/siRNA/replica were imaged. The final result was calculated from 28,800 cells for a single siRNA on average.

### 3.2. Evaluation of the Assay for Cell Cycle Progression

**Figure 3:**
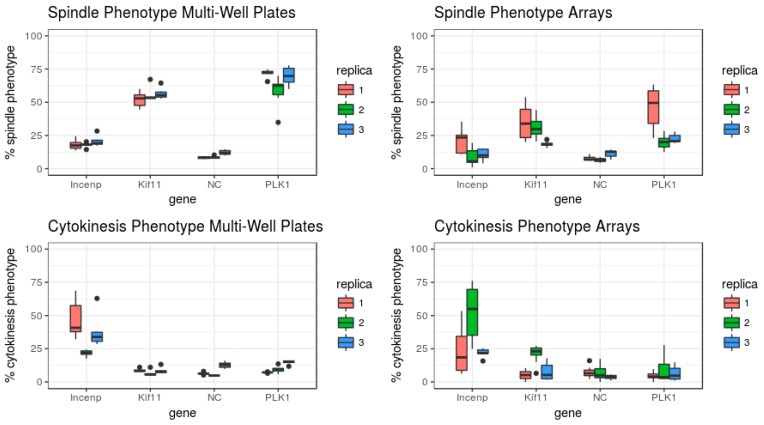
Boxplots of the data obtained from the cell cycle assays on cell arrays and multi-well plates. The horizontal lines in the boxes indicate median values, boxes indicate distribution of 50% of the values of the population, black dots indicate outliers.

In cell arrays and 384-well plates, both phenotypes were significantly distinct from the negative control across all measured replicas under the conditions of RNAi-mediated depletion of the relevant genes (Figure 3 and Table 2). Particularly, well expressed was the spindle phenotype following the depletion of PLK1 and KIF11. Further, the down-regulation of KIF11 and PLK1 was essentially not inducing the cytokinesis phenotype (lower graphs in the Figure 3) and the down-regulation of INCENP caused little appearance of the spindle phenotype. The negative control performed in a similar fashion on both platforms, resulting in 8.6% of cells showing the spindle phenotype on the arrays and 9.7%—on the multi-well plates. In the measurements of the cytokinesis phenotype 5.9% of cells transfected with the negative control on the cell arrays showed the altered shape of the nuclei and 8% of such cells were found on the multi-well plates (Figure 3, Tables S1 and S2).

The appearance of the spindle phenotype following the depletion of KIF11 and PLK1 measured on the multi-well plates is more frequent and less variable than on the cell arrays, so the difference to the results of the negative control becomes clearer (Table 2) in this set-up. In contrast, the percentage of cells showing the cytokinesis phenotype obtained by RNAi of INCENP on both platforms does not differ much. The correlation between the replicates is also stronger on the multi-well plates: Pearson *p* = 0.95 for the multi-well plates compared to *p* = 0.55 for the arrays when measuring the spindle phenotype, and *p* = 0.82 for the multi-well plates compared to *p* = 0.59 for the arrays when measuring the cytokinesis phenotype. We, furthermore, compared the variability among the three replicates: the multi-well plates outperformed the cell arrays in six out of the eight measurements (scoring of two phenotypes, when three genes were targeted and the performance of the negative control in both phenotypes). However, to get significant results on differences in variability, more than three replicates would be needed. We also averaged the intra-plate variance over the three replicates and compared it. We found the intra-plate variance to be higher in the experiments on the cell arrays. This holds for every individual measurement, and overall the difference is significant in three out of the eight cases (*p*-values: 0.027, 0.016, 0.006).

**Figure 4:**
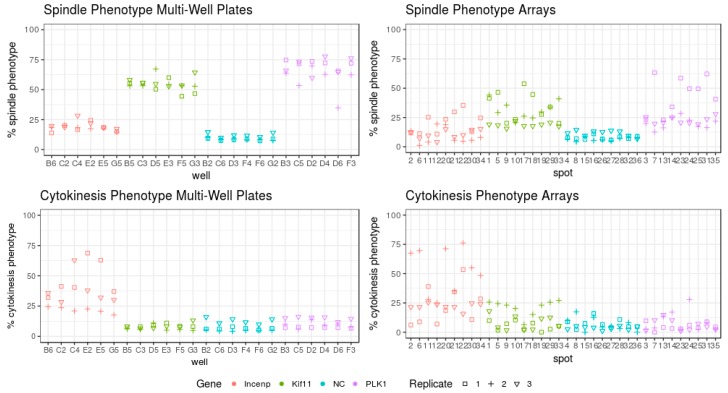
The frequency of the spindle and cytokinesis phenotypes shown in individual wells and individual spots in all three analysed replicates for both formats. The percent of the phenotype frequency in each well is the average of all cells acquired in 9 images/well. Phenotypes induced by the transfection of the chosen siRNAs are indicated in colours (red—cytokinesis phenotype by RNAi of INCENP, green and violet—spindle phenotype by RNAi of KIF11 and PLK1, respectively, and blue—negative control). The individual replicates are indicated by different shapes (replicate 1—squire, replicate 2—plus and replicate 3—triangle).

Besides the comparison on a basis of the individual replicas, we also compared the data on well-to-well and spot-to-spot basis (Figure 4). The spindle phenotype on the individual wells in multi-well plates was less variable than that on the individual spots and clearly better separated from the negative control. The appearance frequency of the spindle phenotype in the individual wells in all three replicas on multi-well plates was remarkably uniform. In contrast, the frequency of the cytokinesis phenotype showed a similar dispersion on the individual spots and wells. 

Impairment of the cell cycle progression is also reflected by the reduced cell numbers following the transfection. For this analysis, we have chosen two replicas out of three per each format which showed the most similar cell numbers with the negative controls (replicas 1 and 3 for cell arrays and replicas 2 and 3—for the multi-well plates (Tables S1 and S2)). Indeed, 40–50% less cells are recorded under the conditions of KIF11 and PLK1 down-regulation in the multi-well plates when compared with the negative control (Table 3). In agreement with the mitotic phenotype frequency (Table 2), 20–30% less cells were recorded on cell arrays, indicating a milder response on this platform.

### 3.3. Evaluation of the Epidermal Growth Factor Endocytosis Assay

**Figure 5:**
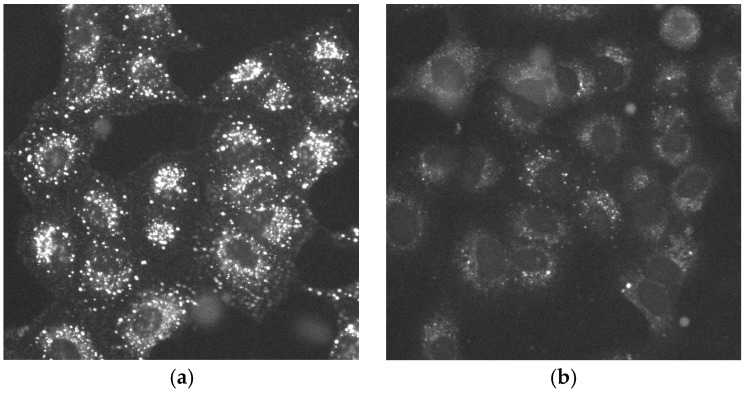
Epidermal Growth Factor (EGF) internalization assay. EGF-Alexa 488 is localized to punctate structures and perinuclear clusters following 20 min of internalization (**a**). RNAi-mediated down-regulation of EGFR inhibits intracellular entry of EGF (**b**). The images were generated in 96-well-plates, but the identical performance was observed and measured in cell arrays.

Next, we have tested the performance of multi-well plates and cell arrays in an additional assay that is independent from the cell cycle. We choose to measure the internalization efficiency of fluorescently labelled EGF. Upon binding to its receptor at the cell surface [33], EGF enters the cells via clathrin-dependent endocytic pathway if the concentration of the ligand is low [34]. Upon the entry, EGF is transferred through the endocytic pathway, where it can be visualized as small punctate structures and larger perinuclear clusters. Depletion of EGFR prevents efficient EGF entry and little EGF-specific fluorescence is recorded under these conditions (Figure 5). The assay is well established and is widely used in the lab scale [9,35] and screening experiments [36]. In contrast to the cell cycle assay, we here depleted only the EGFR, but with 3 different siRNAs. Each of the three siRNAs was positioned on the arrays five times, and three independent replicas of the assay were performed (Table S5). Collectively, 15 images from one array were used to quantify inhibition of EGF endocytosis. Here, 93 cells/spot could be imaged on average, resulting with 465 cells/siRNA/replica (Table S5). 

In general, the assay is fairly easy to perform, but requires several steps of liquid exchange (see Section 2), like changing the media for starvation. These steps turned to be fairly inefficient in 384-well plates and sometimes induced cell loss. For this reason, we have performed the assay in 96-well plates. One well/siRNA was coated and 25 images/well acquired (Table S6). Collectively, 75 images from one plate were used to assess the RNAi-mediated changes. Higher cell numbers were imaged in this set-up: nearly 2500 cells/well/siRNA/replica were collected. 

**Figure 6:**
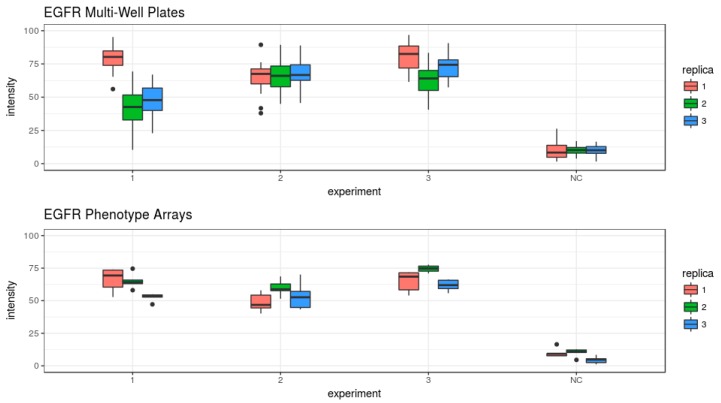
Boxplots of the data obtained from the EGF internalization assays on cell arrays and multi-well plates, grouped by the replicates and siRNAs. The data representation is the same as described for the Figure 2.

Data quantification revealed that the down-regulation of EGFR reduced EGF uptake by 60–70%. All three siRNAs performed in a fairly comparative fashion in both platforms. Also, in this case, the effect of RNAi was significantly distinct from the negative control on both platforms (Figure 6). RNA interference of EGFR did not significantly reduce cell numbers when compared to the negative control in both platforms (*p* > 0.6) (Tables S5 and S6).

In contrast to the cell cycle progression assay, the EGF internalization assay was more robust on cell arrays than in the multi-well plates (Table 4). The correlation between the replicates is also higher on the cell arrays: Pearson *p* = 0.92 for cell arrays compared to *p* = 0.82 for the multi-well plates. 

**Figure 7:**
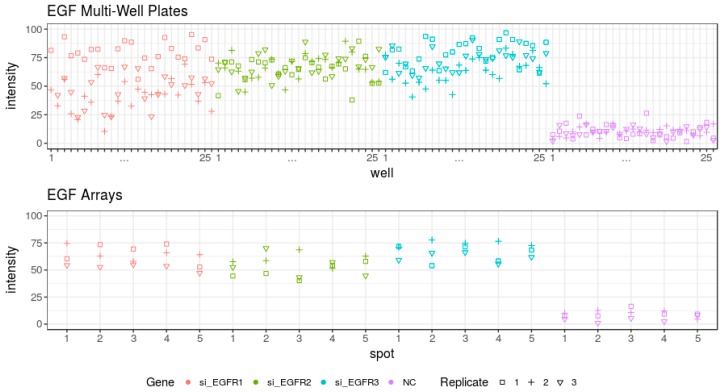
Epidermal growth factor internalization under the conditions of RNAi of EGFR in the individual wells and individual spots in all three analysed replicates in both formats. Cell phenotype in every image taken/per well is shown separately. The colours indicate the performance of different siRNAs (red—si-EGFR1, green—si-EGFR2, blue—si-EGFR3 and violet—negative control). The different shapes indicate individual replicates (replicate 1—squire, replicate 2—plus and replicate 3—triangle).

Similar to the cell cycle assay, we analysed phenotype frequency in individual wells and spots (Figure 7). Epidermal growth factor internalization was more robust over all replicas and spots in cell arrays. Collectively, the results indicate that the type of assay and considered phenotype might be a deciding factor in choosing one or another screening platform.

## 4. Discussion

In order to compare cell-based assays performed in cell arrays and multi-well plates, we focused on two well-established and easy-to-readout assays which enable easy detection and quantification of changes occurring in two essential cellular processes: cell division (Figure 1 and Figure 2) and endocytosis (Figure 5). We performed both assays using reverse transfection in multi-well plates and cell arrays and the images were acquired using the same resolution. The protocols of the siRNA transfection are comparable on both platforms (see Section 2), and their fabrication was done at the same time with the same batches of reagents to ensure data comparability. The data in both assays and in both platforms were generated in independent replicates at different days (Figure 3, Figure 4, Figure 6 and Figure 7). Both platforms enabled to generate RNAi data with the appearance of the expected phenotypes significantly distinct from the negative controls. No well or spot position-dependency could be recorded in our experiments.

The cell cycle progression assay performed better on 384-well plates (Table 2 and Table 3) when comparing various parameters among the replicates (Figure 3). We therefore asked how many replicates would be needed to obtain comparably robust results with the cell arrays as with the multi-well plates. To address this question, we compared standard errors on both platforms. The standard error is a measure of precision of the estimator of a mean, hence here describes how robust the mean values of the phenotypes on the two platforms are. To achieve the same standard error for arrays as for multi-well plates, we would need replicates, where Var_a_ and Var_w_ denote the variances of the data of the cell arrays and multi-well plates, respectively and n_w_ is the number of replicates used for of multi-well plates. Therefore, the multiplication factor is the quotient of the variances. As the variance was constantly higher on the cell arrays in the cell cycle progression assay, more replicates are recommended. However, taking into consideration that the experimental set-up was different and reflects the configurations which perform well in our laboratory, a direct comparison, based on the number of replicates, is difficult and inaccurate. Instead, we compared the number of required images taken under the same conditions. We excluded the replicates with lower cell numbers in the negative control and chose 6 spots on each of the remaining two replicas on the cell arrays as well as six images on the two replicates of the multi-well plates, each with comparable cell numbers. We analysed the appearance of the cytokinesis phenotype, as it turned out to be relatively stable. Comparing the variability between all of the considered images, we found that five individual experiments (e.g., spots on cell arrays) will be needed to achieve the same accuracy of results as in four wells (one image/well) on the multi-well plates. We have not compared the data with the spindle phenotype that way due to fairly different frequencies of the phenotype appearance (Table 2):

$$n_{a}=\frac{{\mathrm{Var}}_{a}}{{\mathrm{Var}}_{w}}\cdot n_{w}.$$

In contrast to the assay of the cell cycle progression, both platforms performed similarly in the EGF internalization assay (Table 4). Here, we also compared the EGF internalization on both platforms using standard deviations and considering one image as one individual experiment. We compared all five spots versus five randomly chosen image positions/well across three replicas. All 5 chosen images out of 25 taken images within one well were spatially distributed and none of them was in direct neighbourhood. Cell arrays performed better with all three siRNAs used. A similar variance to EGF internalization in 15 images in the multi-well plates could be reached by using 6–13 spots on cell arrays. More robust performance of the assay over all spots in all replicas could be achieved in comparison to well-to-well performance of multi-well plates (Figure 7). 

The observed differences indicate that the choice of the platform strongly depends on the type of the assay. For instance, the entry of EGF into HeLa cells is fairly effective, so is the response of the cell to the depletion of EGFR 48 h post-transfection (Figure 5) [36]. As a result, all cells in the population treated with the negative control or siRNAs targeting EGFR can be used for data quantification. The absolute cell numbers which were acquired and quantified in the multi-well plates were more than five times larger than on cell arrays (Tables S5 and S6). Surprisingly, cell arrays were performing better even with a considerably lower absolute cell number. Our data indicate that an assay in which all or nearly all cells in the population show the same phenotype at the time of measurement is well compatible with cost- and work-efficient cell array format (Table 1). Such group of the assays might be measurements of the morphology of intracellular organelles, such as Golgi complex or endolysosomal membranes [36,37,38,39]. Dynamic changes of the organelle size, shape and distribution occurring upon applied treatments (e.g., disassembly of the Golgi complex upon application of brefeldin A) [40] would be also suitable for the cell array format. The same can be said about localization changes of individual proteins (e.g., accumulation of LC3-II (microtubule-associated proteins 1A/1B light chain 3B) on autophagosomes when acidification of lysosomes is blocked) [41]. The labelling of the desired structure could be achieved by antibody-mediated immunofluorescence staining, stably expressed proteins which are tagged to fluorescence proteins or by introducing a reporter by efficient viral infection, such as recombinant adenovirus infection of HeLa or mouse 3T3 cells [6,37]. For instance, cell arrays were utilized in a genome-wide screen of the conventional secretory pathway using adenovirus encoded vesicular stomatitis virus glycoprotein (VSVG) as a model cargo [6]. Furthermore, cell arrays are more beneficial for complex assays which comprise numerous handling steps. For instance, screens analysing proteins which are needed for the intracellular accumulation and activation of integrins require several changes of the media, therefore, consequently, were performed on cell arrays by different groups [5,7]. 

Cellular assays which generate diverse phenotypes would be more robust on the multi-well plate format due to the availability of much more cells. For instance, the shape of *Drosophila* cells can be grouped into five different classes and the down-regulation of PTEN (phosphatidylinositol 3,4,5-trisphosphate 3-phosphatase and dual-specificity protein phosphatase) reduce this shape heterogeneity [42]. In this study, an impairment of early mitosis with siRNAs targeting KIF11 and PLK1 induced two different phenotypes during the assay: interphase nuclei and stalled mitosis with the formation of monoastral mitotic spindle [4,43]. It is not a surprise, therefore, that multi-well plates generated less variable data than cell arrays (Table 2, Figure 3 and Figure 4) as higher numbers of cells (more than seven times) were analysed in multi-well plates than in cell arrays (Tables S1 and S2). Synchronization of the response (e.g., cell cycle synchronization) or life-cell imaging over time might be useful to reduce the data variability without a significant increase of the number of replicas if planning to run such type of assays on cell arrays.

Furthermore, only a sub-fraction of HeLa cells is undergoing division in non-synchronized cultures at a given time. As we measured nuclei changes 24 h after the transfection, a large fraction of cells in the population would have entered mitosis [4]. That explains an accumulation of the early mitotic phenotype following KIF11 or PLK1 depletion (Figure 3). Early mitotic arrest starts to manifest ~18 h after the transfection and peaks at 22–30 h following the transfection with the gradual reduction at the later time points [43,44]. The formation of monoastral mitotic spindles reached a frequency of around 30% on cell arrays in our experiments (Table 2) and was similar to that reported previously on this format and at this time point [4]. Higher phenotype appearance (50–60%) was recorded with multi-well plates in our study (Table 2). This may be due to differences of formulation of reverse transfection reactions between the experimental formats or variations in cell proliferation rates resulting in varying peak times of the expected phenotypes. In contrast, the post-mitotic cytokinesis phenotype following INCENP depletion accumulates later, in a range of 32–50 h [4]. The phenotype peak is not yet reached at the 24-h time point, and in this situation, the percentage of multi-nucleated cells in multi-well plates and cell arrays is fairly comparable (Table 2, Figure 3 and Figure 4). 

Multi-well plates and cell arrays possess their weak and strong sides and the decision which platform might be more beneficial for large-scale experimentation depends upon many circumstances: available infrastructure, funds and cells, the type of the screen (genetic, chemical or combinatorial), RNAi-mediated or CRISPR-Cas9 based gene down-regulation. One of the major parameters is the type of the considered assay. Our data presented here indicate that assays with uniform phenotypes can be performed on cost- and work-efficient cell arrays. In contrast, assays with simultaneous appearance of multiple phenotypes will be more robust on multi-well plates. The decision can be made in the screen optimization phase by rigorous testing of the performance of well-chosen positive controls, timing and the resolution level of microscopy-based read-outs. At last, but not least, statistical data evaluation could be implored in the pre-screen phase to set the optimal conditions and to enhance the output.

## Figures and Tables

**Table 1:** Feature comparison between cell arrays and multi-well plates.

Feature	Cell Arrays	Multi-Well Plates
High-throughput	yes	yes/no
Genetic screens	yes	yes
Chemical screens	yes/no	yes
Reverse transfection	yes	yes
Direct transfection	yes/no	yes
Assay performance needs robotics	no	yes
Edge effects	no	yes
Complex assays can be performed	yes	yes/no
Assay can be performed in cells which are available in small numbers	yes	no
Small amounts of the reagents are required for screening	yes	no
Cell migration can be analysed	no	yes
Cells with the extended shape (e.g., neurons) can be analysed	no	yes
Many cells are available for statistics from one replicate	no	yes
Possibility to cross-contaminate the samples	yes/no	no

**Table 2:** Percentage and standard deviation of cytokinesis and spindle phenotypes on multi-well plates and cell arrays under the condition of RNAi of KIF11, PLK1 and INCENP and the related *p*-values of the Games–Howell test against the negative control. Mean phenotype values are obtained from three independent replicas.

Comparison	Well-Plates		Cell Arrays	
	Mean Phenotype	*p*-Value	Mean Phenotype	*p*-Value
**Spindle phenotype**				
NC	8.6 ± 1.2%		9.7 ± 1.9%	
KIF11	54.8 ± 5.3%	6.9 × 10^−15^	28.3 ± 7.4%	2.6 × 10^−9^
PLK1	66.4 ± 7.6%	1.6 × 10^−15^	29.2 ± 7.9%	5.1 × 10^−7^
INCENP	18.9 ± 3.3%	1.5 × 10^−9^	13.4 ± 6.6%	3.9 × 10^−2^
**Cytokinesis phenotype**				
NC	5.9 ± 1.2%		8 ± 3.9%	
KIF11	7.8 ± 2%	1	11.5 ± 5.6%	3 × 10^−2^
PLK1	10.5 ± 1.5%	2 × 10^−1^	6.6 ± 5.4%	9.7 × 10^−1^
INCENP	35.5 ± 10.1%	2.6 × 10^−6^	32.7 ± 13.1%	1.8 × 10^−6^

**Table 3:** Percentage of the remaining cells on multi-well plates and cell arrays following the depletion of KIF11, PLK1 and INCENP. Mean values of cell numbers are obtained from two independent replicates.

Comparison	Well-Plates		Cell Arrays	
	Mean Cell Numbers	*p*-Value	Mean Cell Numbers	*p*-Value
NC	100%		100%	
PLK1	49.6%	9.9 × 10^−9^	68.2%	1.2 × 10^−2^
KIF11	58.9%	8.2 × 10^−7^	73.7%	2.2 × 10^−2^
INCENP	59.2%	4.6 × 10^−4^	93.1%	9.1 × 10^−1^

**Table 4:** Mean intracellular levels of EGF following down-regulation of EGFR on multi-well plates and cell arrays and the related p-values of the Games-Howell test against the negative control. Mean values of the intracellular EGF signal and standard deviation values were obtained from three independent replicates.

Comparison	Well-Plates		Cell Arrays	
	Mean Intracellular EGF Signal	*p*-Value	Mean Intracellular EGF Signal	*p*-Value
NC	89.8 ± 5%		91.8 ± 3.3%	
EGFR_1	44.4 ± 12.8%	8.8 × 10^−14^	38.7 ± 6.6%	4.1 × 10^−14^
EGFR_2	33.5 ± 10.8%	1.5 × 10^−14^	45.9 ± 8.4%	8.8 × 10^−14^
EGFR_3	28 ± 10.8%	4.4 × 10^−10^	33 ± 5.5%	4.7 × 10^−14^

## Supplementary Materials

The following are available online at http://www.mdpi.com/2571-5135/7/2/13/s1. Table S1: Cell cycle assay on cell arrays, Table S2: Summary of the cell cycle assay in 384-well plates, Table S3: Frequency of the spindle phenotype in 384-well plates shown in every image, Table S4: Frequency of the cytokinesis phenotype in 384-well plates shown in every image, Table S5: EGF internalization assay on cell arrays, Table S6: EGF internalization assay on 96-well plates.
